# Involuntary Resistance

**DOI:** 10.1007/s10767-023-09442-5

**Published:** 2023-03-23

**Authors:** Mikael Baaz, Mona Lilja, Malin Wallgren

**Affiliations:** 1 University of Gothenburg, Gothenburg, Sweden; 2 School of Global Studies, University of Gothenburg, Gothenburg, Sweden; 3 School of Business, Economics and Law, University of Gothenburg, Gothenburg, Sweden

**Keywords:** Resistance, Covid-19, Neoliberalism, Norms, Governing

## Abstract

This paper problematizes the notion of “intent” through the concept of “involuntary resistance”. Departing from the narratives of employees in nursing homes in Sweden during the Covid-19 pandemic in 2020 and 2021, we suggest that neoliberal norms and a local management that capitalizes on social hierarchies (sex, age, class, etc.) were the context of the strong biopolitical state management that occurred due to the Covid-19 pandemic. The friction between different forms of governing became a seedbed for an involuntary resistance with an unclear intent against the state recommendations. This sheds light upon the need to (re)frame the current dominance of specific types of knowledge that are constructed in the field of resistance. We suggest that new paths of thought are needed—within social sciences—that work towards a wider conceptualizing of resistance, which embraces practices that lie outside the common thought of dissent.

## Introduction

This paper rethinks the concept of “intent”, through identifying a pattern of involuntary resistance that took place in nursing homes in Sweden during the Covid-19 pandemic. A “common story” within social sciences depicts resistance as a social practice that is carried out by organized resistance actors who have a clear intent against defined and known power-relations. Or as pinpointed by Sherry B. Ortner (1995, p. 174), resistance has previously been understood as: “a relatively unambiguous category, half of the seemingly simple binary, domination versus resistance. Domination was a relatively fixed and institutionalized form of power; resistance was essentially organized opposition to power institutionalized in this way”. While the narrative presented by Ortner draws tight boundaries for what can be counted as dissent, processes of globalization, modernity, new forms of liberalism and changed capitalism have recently complicated the storyline of resistance. With societal transformations, power has been changing and, thereby, so has resistance; Fordist capitalism evoked one kind of resistance while the engagement with, for example, neoliberal capitalism under the pressure of transnationalism, unleashes other forms of dissent. Thus, with the shift to more complex relations of governing, the repertoire of resistance practices has expanded and changed in form (Lilja, 2022; Lilja & Vinthagen, 2014; Mumby et al., 2017).

The above implies that contemporary resistance practices need to be understood as products of shifting economic, political and cultural topographies of power—not least during the Covid-19 pandemic (Mumby et al., 2017). In this paper, *we interrogate the “involuntary” resistance, which was provoked by multiple and interacting forms of power during the Covid-19 situation, with a focus on the question of intent.* We suggest that “resistance”, as an analytical concept, has a lot to gain from including a wider range of practices other than only those that are intentional, deliberate and planned. Among other things, the resistance of the healthcare workers we interviewed in 2021 could be understood as an effect of frictional processes of governing/management; however, there was still no intention to resist power. Or in other words, although the healthcare workers chose to defy the state recommended measures, which required them to stay at home if they had symptoms of fever, myalgia, fatigue, runny nose and/or dry cough, they did not intend to resist. Thus, when obstructing and breaking the regulations, our respondents displayed intent, but not the intent to resist.

Summing up, in the analysis below, we elaborate on the notion of “involuntary resistance” that includes an unclear intent through a qualitative case study of the Covid-19 situation in Sweden during 2020 and 2021. Here it is important to note that the paper does not aim to extend the findings of the study to the Swedish population at large. Our qualitative research data does not provide support for generalizability if it is defined as “[t]he extent to which you can come to conclusions about one thing (often, a … population) based on information about another (often, a … sample)” (Vogt, 2005, p. 131). Still, our in-depth interviews provide us with data from which to theorize specific paths of dissent.

The resistance we identify will also be placed in relation to, and is understood through, the context that the Covid-19 pandemic and contemporary relations of power in Sweden offered us. We suggest that neoliberal norms and a local management that utilized social hierarchies (sex, age, class, etc.) were the context of the strong biopolitical state management that occurred due to the Covid-19 pandemic. The friction between different forms of governing must be localized and embraced to understand resistance that is situated outside the common thought of dissent.

To understand the micro-study that we conducted, we also want to shed light upon the situation in many nursing homes in Sweden during the pandemic. Sweden had high death rates among elderly people who were living in long-term residential care facilities during that time—at least in the early stages of it. For example, in 2020 the mortality rate in nursing homes was 23% higher compared with the 2016–2019 average, 14% higher among the elderly with home care and 13% higher among the elderly outside elderly care (Szebehely, 2020). As stated by former prime Minister Stefan Löfven, Sweden “did not manage to protect the most vulnerable people, the most elderly, despite our best intentions.” Thus, the caretaking in nursing homes during the pandemic exhibited serious, even shocking, failures, which makes the site a particularly interesting case for interrogation.

## Methodological Considerations

To explore the involuntary resistance that occurred during the Covid-19 pandemic in Sweden, we have been studying various secondary sources, primary scholarly literature, texts by stakeholders to the event(s) and TV and radio documentaries, as well as primary sources, such as Government Committee’s reports, opinions and (amendments to) legislation. In addition, we have carried out five in-depth interviews with healthcare staff members, three of whom were employed by the hour (often offered work by the usage of text messages) and two assistant nurses, who also worked as union officials, in retirement homes in Sweden. Of the five respondents four were self-identified women, one self-identified man and one immigrant.^1^ Together, the above combination of data has provided us with differentiated material that contains different, often complementary stories, from which we have identified the resistance that is addressed in this paper.

As stated above, the ambition of this paper is not to give an extensive and general image about resistance in Sweden, but, rather, qualitative data is used to theoretically engage with a specific form of resistance. Thus, the aim is not to illuminate patterns or generalize regarding the larger Swedish population. As Peter Esaiasson et al. (2017) point out, while qualitative research interviews are not representative of all similar cases, they can still provide a base from which it is feasible to theorize *possible* and existing forms of subversive actions. Theories on how resistance has been, as well as can be, enacted can be constructed from selected empirical accounts. Or, in the words of Robert K. Yin (2009, p. 15), “… case studies, like experiments, are generalizable to theoretical propositions and not to populations or universes…in doing a case study, your goal will be to expand and generalize theories (analytic generalization) and not to enumerate frequencies (statistical generalization).” What Yin points towards in the quotation, when applied to the context of this micro-study, is the possibility of building a theory of resistance from the experiences of the hourly employed or their union representatives during the Covid-19 pandemic, while not giving a full picture of the pandemic itself.

## Sweden’s Covid-19 Policy and Governing through Self-management

Sweden’s favoring of what Katarina Giritly-Nygren and Anna Olofsson have called a “soft lockdown” as a response to Covid-19 involved individual strategies of self-management in order to govern the pandemic situation (Giritli-Nygren & Olofsson, 2020, 2021. See also Fredriksson & Hallberg, 2021. cf. Lindström, 2020; Winblad et al., 2022). In the early stages of the pandemic, the Swedish government classified the coronavirus as “dangerous to society”. While this classification made it possible to order mandatory quarantine in cases where recommendations were not followed, many of the general guidelines were provided as recommendations without coercion.

As the pandemic started, the public was urged to stay at home if having symptoms; thereafter, there were recommendations to shift to remote working or teaching. More restrictive measures were implemented for businesses serving food, and people were advised against any non-essential travel. A ban on public gatherings of more than 500 persons was followed by recommendations for even smaller audiences. In addition, a ban on visiting retirement homes was implemented to safeguard the elderly, high-risk population (Andersen et al., 2022).

One of the most debated aspects of the pandemic response in Sweden was the time it took to increase the Covid-19 testing capacity. During the first half of 2020, testing capacity in Sweden was limited and reserved to protect the most vulnerable in society. This was partly because the Public Health Agency of Sweden suggested that there was no medical reason to test members of the public who fell ill with Covid-like symptoms. Moreover, the multi-level governance of Sweden fell short of providing large-scale testing (Fredriksson & Hallberg, 2021). The Swedish healthcare system is highly decentralized. The regions are self-governing with politically elected assemblies, as set down in the Swedish constitution, while the *state* is responsible for overall healthcare policy and laws (Fredriksson & Hallberg, 2021). This further complicated the implementation of the large-scale testing.

The above indicates that, instead of favoring the use of force, governing to a large degree took place by using different “biopolitical” and “ethopolitical” strategies (cf. Foucault, 1978; Rose, 2001; Giritli-Nygren & Olofsson, 2020, p. 1031, 2021). In other words, the state strategy mostly leaned on “recommendations”, “solidarity” and “common sense”, formulated on the basis of the collected statistics. The Swedish Public Health Agency (*Folkhälsomyndigheten*) closely monitored the Covid-19 situation by carrying out microbiological analyses and risk assessments while mapping the development of the outbreak; thereafter, reports were published on their homepage to be “known” by the public. Governing also took place through mapping how people moved, while simultaneously instructing people how to behave “if you are planning to travel” and when “Using public transport”.^2^

Overall, the Agency worked systematically to prevent the spread of infection in society, by encouraging self-management. Or as stated on their homepage: “The Public Health Agency of Sweden’s regulations and general guidelines relating to everyone’s responsibility to prevent Covid-19 infections.”^3^ The managing was very specific and detailed, and regulated people into their “bare life” (Agamben, 1998). For example, in a “fact sheet” named “How to wash your hands”, there were detailed instructions on how people should wash their hands.^4^ In addition to these kinds of directives, the Swedes were, as stated above, requested to uphold social distancing, self-isolation and work from home (Jonung, 2020). In a speech to the nation in November 2020, the (former) Prime Minister Stefan Löfven said the following:

A stranger you infect could get very ill. A friend you infect could need care. A grandparent you infect could die. These are the people for whom you should make sacrifices. These are the people for whom you have to show decisiveness, self-discipline and a sense of responsibility. When we are through this crisis, everyone should be able to remember how we helped each other. Remember the solidarity. Remember the feeling of community and the feeling of doing the right thing. Remember how we pushed the spread of infection down and raised our country. But then we have to show our unity and our sense of responsibility here and now. And that it is stronger than the virus we must defeat. Let’s do this now. Together. For Sweden. (Löfven, 22 November 2020)^5^

Löfven emphasized that there should be no exceptions to this (or any other) general recommendation(s); the message was simply that everyone had to “discipline” themselves for the sake of others: “Nobody should take chances. Not one of us can go to work with symptoms. Young, old, rich, or poor does not matter—everyone needs to do their part” (Löfven, 22 March 2020).^6^

Moreover, the techniques of controlling citizens also involved alluding to Swedes as being “rational”. In a radio program, the Swedish state epidemiologist, Anders Tegnell, stated that: “I think what we are talking about here is the Swedish culture, how Swedes interpret recommendations from the authorities … I think most people see it as very clear advice on how to do this in the best possible manner …” (Radio Sweden, 27 March 2020).^7^ According to Tegnell, people understood that the recommendations given by the governing bodies were the ones considered to be the best for them. Here, we suggest that the state epidemiologist—by assigning the population “agency” and freedom of thought—entrusted the (individual) citizen to make the “right decisions”; that is, to act in line with the “recommendations” that were being made. This is a form of governing and use of power that targets everyday life, which works at the level of ongoing subjugation, governs our gestures, dictates our behaviors, et cetera. It is a kind of “emotional politics” that is launched to create or, perhaps more correctly, elaborate disciplined citizens, by constructing them as “free”.^8^ Such measures, implemented by the state apparatus, aimed to constitute the subject gradually, progressively, and materially (see further, Foucault, 1982; Rose, 2000).

However, not only “common sense” but also solidarity was pictured as an engine for following the national recommendations. Then Minister of Culture Amanda Lind (The Green Party), when debating culture in the Swedish parliament on 27 March 2020, stated:

Madam President! It is our duty as a society to do everything in our power to protect those who are at risk of harm. As the Prime Minister said in his speech to the nation, there will be “a few crucial moments in life when you have to make sacrifices, not only for your own sake but also to take responsibility for your surroundings, for your fellow human beings and for our country. That moment is now. That day is here. And that task applies to everyone”. (…) It is good. These are resources that come in handy in a moment like this. But we also have something else in store: a reserve of human trust, solidarity, and consideration. It is from this gold reserve of human care that we now pick out the power in our society, in the moment when it is most needed.^9^

By constructing the Swedish people as compassionate, the citizens are encouraged/required to discipline themselves; they should voluntarily wash their hands, stay at home, et cetera. Regarding this, one of our respondents said this:

I think that in these political speeches it has been very much like this … You must feel solidarity with the Swedish people. You must not go to work when you are sick. We must show solidarity with each other. Everyone must stay at home.^10^

This strategy can be understood by what Nikolas Rose (2000) has labeled “ethopolitics”; that is, “the emergence of a technology of governance that seeks to regulate individual conduct with reference to dominant moral discourses of responsible behaviour” (McKee, 2011, p. 3400; Giritli-Nygren & Olofsson, 2020). It is this kind of governing that was put in place by Löfven as he encouraged citizens to “make sacrifices” to save “a stranger” or “a grandparent”. This governing strategy could be considered “a project of rule that seeks to govern the ethical self-regulation of individuals in terms of fixed moral codes and aesthetic choices” (McKee, 2011; Rose, 2001, p. 1399): Rose writes:

By ethopolitics I mean to characterize ways in which the ethos of human existence—the sentiments, moral nature or guiding beliefs of persons, groups, or institutions—have come to provide the “medium” within which the self-government of the autonomous individual can be connected up with the imperatives of good government. (2001, p. 18)

Rose concludes: “If discipline individualizes and normalizes, and biopower collectivizes and socializes, ethopolitics concern itself with the self-techniques by which human beings should judge themselves and act upon themselves to make themselves better than they are” (Rose, 2001, p. 18; Lilja & Baaz, 2022). Drawing on the above, we would like to propose that the Swedish pandemic strategy was partly built on insisting on certain moral guidelines, which strive to provide the population with the “proper” directions of self-management, and partly on the construction of “rational” and “free” citizens, who happily and voluntarily adapt to the rational recommendations of the state representatives (cf. Giritli-Nygren & Olofsson, 2020). By this, let us now turn to a discussion of neoliberal governing and precarization before we continue with our case study and analysis.

## Neoliberal Governing and Precarization

Above we have discussed ethopolitics as a form of state governing; however, this governing prevailed side-by-side with other forms of power during the pandemic—for example, the neoliberal management and disciplinary power that was encouraged or employed by both municipal and state actors (see e.g., Rådestad & Larsson, 2018; Peck, 2010; Miller & Rose, 2008; Bevir, 2016; Brown, 2015). Neoliberalism can be viewed as both a product and a critique of biopolitics; it is considered both a practical way of governing (dependent upon biopolitical domains) as well as being concerned with too much governing (cf. Dean, 1999, pp. 118–210; Harvey, 2005; Hamann, 2009; Brown, 2015; Springer, 2010, 2016).

Sweden today—despite its reputation for egalitarianism and social democracy, like many other countries—is marked by a global neoliberalization, which is manifested by growing social and economic inequality and a wider separation between classes (Holmqvist, 2021). As stated by Mikael Holmqvist (2021, p. 1356): “A market-based way of organizing society characterizes most sectors, including healthcare, pensions, and education; the private corporate sector dominates the labor market morally and socially by idealizing deregulation and entrepreneurship—as a result, ‘new public management’ is a widespread phenomenon”. This politics has led to entrenched inequalities in Sweden. Between 1985 and the early 2010s the growth in inequality increased by one-third and was the largest among all OECD countries. This means in practice that Sweden’s richest 1% of earners saw their share of total pre-tax income nearly double; it was 4% in 1980 and 7% in 2012.^11^ The percentage of persons at persistent risk of poverty has also increased in recent years. In 2020, just under 15% of the Swedish population were at risk of poverty and nearly two-thirds of those people were at persistent risk of poverty.^12^ This has gone together with homelessness and housing exclusion, which are increasing problems in Sweden. Between the first homelessness count in 1993 and the most recent in 2017, the number of homeless people had doubled (European Commission, 2019). Child poverty numbers in Sweden are higher compared with neighboring countries. Approximately 12% of children growing up in Sweden are living in what could be defined as child poverty. Children with immigrant parents, and parents who have unusually low levels of education, are strongly overrepresented among the chronically poor (Odenbring, 2019; Lindquist & Sjögren Lindquist, 2012).

Following the widespread adoption of neoliberal ideas, “gig-economies” have started to emerge at many locations around the world as well as in Sweden. In a gig-economy, companies and institutions can maximize flexibility by engaging and paying workers only when they need them to perform specific tasks; by this, they also avoid paying social insurance charges and contractual wages. The gig-economy can be seen as the ultimate neoliberal practice, in which the employer gains freedom from obligations, while the “employee” encounters minimal social security, and experiences financial difficulties in raising a family and buying, or even renting, a home. As is reflected in our interviews, employees in the gig-economy must be on constant alert, ready to work at any time (Heeks et al., 2021). Of the two assistant nurses, who also worked as union officials, one put it like this:

Quite a few say: [L]ook at McDonalds and their policy of sending job offers by text … So what, the municipality of Gothenburg does exactly the same. [People employed by the hour] sleep with their cell phones under the pillow. Alice [a fictitious name] does so. Because, if she is not the first to respond … they send the same text to ten different people. The one responding first will get the gig.^13^

Another respondent suggested that the employees of the gig-economy:

(…) would like to have a permanent job. They want the security. They want to feel that they can take a mortgage and buy an apartment … They want to feel that they can take a vacation … That they can be free also during the summer… They want to be there for their families and know which weekends they must work and all that. [They do not wish] to work every single weekend for four weeks in a row, because that is the only hours you will get. It is a terrible way of life for adults, if I may say so, who have family and children. It is not dignified at all … [They] find it very difficult to get the economy together ... [T]he employer, they [also] give them less hours. If I am supposed to work from 7 am to 4 pm, a replacement is not allowed to work from 7 am to 4 pm but is only allowed to work from 7 am to 2 pm. They only get part-time jobs when they work. They have a very hard time getting up to full time if they do not do more shifts and then they have fewer days off, which also makes them more tired.^14^

The hourly employees not only get fewer hours than those on permanent contracts, but they also have problems with getting compensation if they get sick. In 2019, Sweden introduced a “qualifying deduction” (*karensavdrag*), which means that during each period of sickness, sick pay is subject to a payment reduction that is equivalent to 20% of one’s average weekly sick pay. This *karensavdrag* replaced the previous *karensdag* or “waiting day”, which was the name of the first day of sickness where no benefit is paid. During the coronavirus pandemic, however, the government began covering the cost of sick pay from day one in an attempt make people with symptoms stay at home. According to one of our respondents, however, this change did not impact upon the situation of the so called sms-employees, as their assignments were often for just one day: “we get new temporary workers every day, because if that temporary worker falls ill, the employer must pay compensation for him/her. (…) Therefore, they only book hourly employees one day at a time”.^15^

The economic uncertainty of many who work in the health sector is the result of neoliberal norms and governing, which interact with various discourses of identity. Precarity “denotes structural inequalities—uncertainties that result from relations of domination along with gender, race, ethnicity, class, sexuality, nationality” (Lorey, 2015). This suggests that the neoliberal governing of subjects is not neutral, but builds on, as well as utilizes, various power relations. As expressed in an op-ed piece in one of Sweden’s biggest newspapers:

Both the trade and the health and care sector are women and youth dominated sectors, where the majority work as day laborers, standing with cap in hand, begging and asking for more work shifts to be able to pay their bills. This systematic exploitation of labor must end. That women, young people as well as new Swedes, who often begin their careers in Sweden in our profession, are still treated as second-class labor is astounding, and at the same time so uncomfortably clear.^16^

This quotation indicates that neoliberal discourses and growing gig-economies interact with hierarchical constructions along with gender, race, ethnicity, class, sexuality and nationality; that is, various relations of power, which create uncertain and precarious lives for many people (who work in the care sector). Moreover, as suggested below, during the Covid-19 pandemic, different instruments of Swedish state governing sometimes ended up interacting and creating tension with neoliberal norms and conditions of economic insecurity. In the next-coming sections, we discuss how various forms of involuntary resistance were evoked by the friction between different forms of governing. In line with Anna Tsing (2005, p. 5), we suggest that the concept of “friction” here “reminds us that heterogeneous and unequal encounters can lead to new arrangements of culture and power”.

## Resistance and Intent

Today there is a rich and varied scholarship that embraces the concept of resistance within the social sciences. Much of this scholarship, however, focuses on large-scale and organized forms of collective resistance (civil society, social movements, revolutions and so forth) and demonstrates how resistance is often carried out through non-cooperative forms of resistance, such as demonstrations, sit-ins or riots (see e.g., Chenoweth & Stephan, 2011; Sharp, 1973; Vinthagen, 2015). Another strand of scholarship, contrary to the above, has embraced the theorizing, which was initially introduced by James C. Scott, that focuses on more hidden forms of agency (Scott, 1977, 1989, 1990, 2019). Researchers’ approach to resistance within this field has resulted in more elaborate studies of, for example, peacebuilding or anti-colonial struggles (see e.g., Chandler, 2013; Hardiman, 2018). Inspired mainly by Scott—but also others—the scholarship on everyday resistance has disclosed a whole new landscape of invisible politics; virtually everyone who is subordinated by some power is participating in politics through “mundane or petty acts by circumventing, negotiating, manipulating, or undermining hegemonic power in their family, workplace, or neighbourhood” (Kasbari & Vinthagen, 2020: 1; see also Baaz et al., 2023; Lilja & Vinthagen, 2018; Baaz et al., 2016). According to Scott, those who are powerful have “a vital interest in keeping up the appearances appropriate to their form of domination. Subordinates, for their part, ordinarily have good reasons to help sustain those appearances or, at least, not openly to contradict them” (Scott, 1990, p. 70) Thus, resistance cannot only be understood as an “overt collective defiance of powerholders” in contrast to a “complete hegemonic compliance” but instead, most forms of resistance fall between these two—not least through different kinds of “everyday resistance”, which Scott has interestingly displayed (Scott, 1990).

The novel and rich theorizing by Scott, and the many scholars inspired by him, has considerably added to the scholarly understanding of resistance. The main problem with Scott and many of his followers’ interpretation of resistance, as we understand it, is that it often focuses on resistance against explicit claims (of class interests) and does not recognize unintended or “other-intended” resistance (Lilja et al., 2017). Everyday resistance, according to Scott (2019, p. xi), “succeeds by systematically concealing intentions or, in fact, misrepresenting intentions as loyal and allegiant”. The “intent” of the resister is difficult to distinguish; still, as he maintains in *Domination and the Arts of Resistance: Hidden Transcripts* (1990), it is important to do so to establish what are to be considered as acts of resistance.

Asef Bayat (2000), who has proposed an alternative concept to everyday resistance, namely “quiet encroachment”, argues that:

Scott makes it clear that resistance is an *intentional* act. In a Weberian tradition, he takes the meaning of action as a crucial element. This intentionality, while significant, obviously leaves out many types of individual and collective activities whose intended and unintended consequences do not correspond. (Bayat, 2000, p. 543, italics added)

Bayat describes how many poor families in Cairo or Tehran tap electricity and running water illegally despite their awareness of their criminal behavior. Still, they do not want to express their “defiance vis-a-vis the authorities, but rather they do not find any other ways to acquire them.” (Bayat, 2000, p. 543). By acknowledging this imprecise intention, he proposes that instead of excluding resistance, whose intentions we cannot secure, we should investigate the, sometimes far-reaching, effects of practices of dissent. Thus, in line with us, Bayat suggests that the intent of resistance is sometimes ambiguous, unknown or non-political, yet still qualifies as resistance. Some acts are in themselves a response to power relations but are not being clearly desired or intended. In addition, in studies of resistance as discursive, subversive, mobile and creative points, which are reiterated within strategic fields of relations of power, it becomes less interesting to locate the intent or even the subjects of resistance (Foucault, 1990; Lilja, 2021, 2022; Lilja & Lilja, 2018; Malmvig, 2016). Thus, whether or not intent is relevant partly depends on what kind of power one studies. Below, we further elaborate on the complexity of the mentioned governing and illuminate how this molded our respondents’ acts during the pandemic situation.

## Frictional Governing and “Involuntary” Resistance: The Case of Sweden during the Covid-19 Pandemic

Below, we discuss the notion of “intent” in Resistance Studies by departing from the narratives of union officials and employees in nursing homes during the Covid-19 pandemic in Sweden in 2020 and 2021. Among other things, we suggest that, during the exceptional state of the pandemic, friction between different forms of governing became a seedbed for an involuntary resistance that had an unclear intent against state recommendations.

### Different Systems of Governing, Disciplinary Power and “Involuntary” Resistance

As displayed above, the speech acts of Löfven and Lind as well as the Swedish Public Health Agency, including state epidemiologist Tegnell, have not only constructed “recommendations”, but also suggested optimal behaviors to mobilize an emotional and ethical self-management of the population; however, as became obvious during the pandemic, some sms-employees experienced that there were consequences for following the recommendations. Five assistant nurses described the situation of the substitute employees in an op-ed in one of Sweden’s largest newspapers:

Instead of investing in over-employment to fill in sick leaves, one has instead created a system of “text employees”, who move from unit to unit. This is the worst form of employment, making you feel afraid and/or unable to afford being sick. Our subs are worth their weight in gold and deserve decent employment conditions so that they too can stay at home if they are sick or showing symptoms of infection. … If you are a sub employed by the hour and called in [only] when needed, you have no qualifying day of sickness. To put it bluntly: If you do not work, you have no food to put on the table. Then, choosing to stay home is far from obvious.^17^

Likewise, one assistant nurse, who also worked as a union official, argued:

… If I had been offered three work shifts during the month [so far] and is then offered a fourth one and I am not feeling very well. I know that the salary will be paid out next month, and I only have had four working shifts. What will I do that month? [Considering this], I take the offered shift, even if I realize that I should not. I think … it must be a very … difficult situation for people in situations like this to make the [right] decision. I mean, to decide and thinking: “but, if [something happens]?”. And, at the same time [experiencing] that you really have no choice, because you need to survive … But I believe that the subs have probably also regretted that they have gone working despite not feeling well; however, feeling that not being in a position to make the choice that you really wish to make.^18^

The difficult situation of some “text employees” was confirmed by one of our respondents, a sub employed by the hour, who stated that “people have gone to work with covid because they could not afford to be home sick”.^19^

We would like to propose that the friction captured in our interviews between the governed and those who are governing is produced through various norms and processes of materialization. The regulatory norms proposed by the state—for example, in the public speeches by the Prime Minister—materialized in the many citizens, who worked from home and avoided visiting public places during the pandemic. This materialization prevailed through the reiteration of norms. Still, it was processes of materialization that were never free from friction. As our respondents suggested, in the gig-economy, not all workers have chosen to perform according to the state norms.^20^ As the regulatory norms of the Swedish Public Health Agency materialized, some citizens considered themselves unable to stay at home for economic reasons. This might, at least partly, explain why 40% of the staff in a retirement home in Sweden on 6 April 2020 went to work sick, even though they had been encouraged to not show up if they had symptoms. Four out of the 57 staff members tested positive for Covid-19 (two of which had symptoms).^21^ A recent report showed that during the pandemic, two out of ten in Sweden, who temporarily or more permanently uphold a “working class” position, state that they have worked during the pandemic even though they felt sick, as they could not afford to be at home, or that no one else could do their job if they were away.^22^ Or as expressed by the assistant nurses mentioned above: “From insecure employment follows … that you must be constantly available and do not dare to turn down offered work shifts. This is not good for anyone”.^23^

We would like to suggest that the technology to govern, by seeking to regulate individual conduct with reference to dominant moral discourses of responsible behavior, sometimes failed due to the structural frames of the gig-economy. One of our respondents discussed possible reactions to Löfven’s speech from the perspective of people being employed by the hour at nursing homes; she said:

[If] I am going to imagine what they might think, it might be something like this: “Well, that is easy for you say, being the Prime Minister. You are secure. [But I must] put food on the table, for myself, my children and family”. I think people think like this. I probably would have done so too. … “Well, that is easy for him to say … Well, easy for you to say, thank you very much. I go to work when I feel I should do so”. This is how I probably would have thought.^24^

Another respondent asked why the gig-employees should:

… come and work and care and take responsibility for the whole community and stay at home when feeling sick so that you do not get any money. And why should one be loyal to them, someone who constantly exploits you?”^25^

Considering the above, we would like to suggest that the aim of seeking moral self-regulation of individuals by applying ethopolitical strategies was, sometimes, undermined by a sense of being precarious. The fear of individuals who are employed by the hour involves the future or, perhaps more correctly, projections of the future as being indefinite and threatening. Hence, fear could be considered as an engine of the involuntary resistance that was performed during the Covid-19 pandemic, despite the various disciplinary strategies that were employed by authorities, friends and colleagues alike. Our interviews display the pressure that temporary staff members were exposed to to self-manage themselves in line with the recommendations. One of our respondents stated:

I participated in a radio show… In the show, also someone from the region participated—the person being responsible for disease control … this person thought it was terrible, arguing “how could these people [the one being employed by the hour] expose others to risk?” … I get so tired of people judging others … We are talking about poor people, people who are employed by the hour.^26^

This quotation reveals how different punishments—here in the form of public shame and dislike—were sometimes distributed to those who did not follow the state recommendations. One of the sms-employees stated the following:

Even with mild symptoms, we are supposed to stay home. But it is difficult during the winter because the nose runs when it is seven degrees below zero (Celsius), regardless of whether it is a covid-19 pandemic or not. Then you can only hope that it is not covid-19 … social media has also made it difficult to work in healthcare, as there has been a lot of social pressure [lately]. I am afraid that I will accidentally infect someone. It is more or less forbidden to cough, sneeze, et cetera. Many are on sick leave and the lack of staff puts pressure on the ones at work staff … That has created a lot of mental stress.^27^

What is described above is a situation in which healthcare employees felt monitored on social media by friends and colleagues. The control and shaping of the Swedish population also included forms of “punishment” other than the ones described above. There was, for example, a constant threat from the authorities to apply more and harder restrictions. In addition, at least four people in Sweden have been charged with going to work with an active Covid-19 infection. All of them are women who work in the care sector. For a person to be convicted of “creating danger to another”, it is required that they have acted with “gross negligence”. Until 2022 Covid-19 was, as stated above, classified as a “public health hazard” and several employees around the country lost their jobs when it had been discovered that they defied the infection control rules and had gone to work with Covid-19.^28^

When discussing disciplinary punishments and state governing, it is important to establish an intersectional perspective. As stated above, the Swedish healthcare system has been subject to a neoliberal restructuring where, among other things, cheaper forms of labor have been increasingly sought in order to reduce the cost of providing elderly care (Ahlberg et al., 2019). Subjects marked by categories such as class, gender and ethnicity have increasingly become a pool of cheap labor in healthcare and other welfare sectors.^29^ In regard to this, one of our respondents, a union official, stated:

We have a microscopic few [sms-employees] who could be assistant nurses, but they want to be free and decide themselves when to work. ... Most sms-employees come from other countries, they are maybe 45–50 years old and want to get into the labor market. … Over 80% of those who work in elderly care are women.^30^

All in all, Swedish retirement homes could be seen as populated by different practices, norms and subjects, which are constituted and held together as “neoliberal”. During the pandemic, these neoliberal practices, norms and subjects came, as mentioned previously, to co-exists and interact with a strong ethopolitical state management. In the friction between different forms of governing—strong governmental rule, disciplinary punishments and a neoliberal management of local retirement homes—various forms of involuntary resistance took place. This is further elaborated on below.

### Involuntary Resistance and the Question of Intent

The decision of care workers to go to work, even though they did not feel well, built on various rationales. This was expressed in several of our interviews. For example, one union-official, who also worked as an assistant nurse, told us the following:

You can “choose” not to receive any salary, but then you cannot pay the rent or give your children food or clothes; or you go to work a little snotty and think: This is probably just a little allergy or something like that. There is no harm in that, there is no intent … [T]here was a lot of such [behavior] in the early 2020. Well, but how can they with ... and so on. Well … it is so damn easy for us to sit and say, who have monthly salaries and say [this and] that ... and just carry on like that [The subs] were not even entitled to compensation since they had no booked shifts.^31^

According to this assistant nurse, some who were employed by the hour (re)categorized the runny nose from “covid” to “allergy” rather than calling-in-sick. What she suggests is that competing stories around the “runny nose” gave room for different rationalities that justified presenteeism. The “rationale shopping” was made feasible due to several competing explanations around sniffling. Many of these—for example, pollen allergy, “morning runny nose” or ordinary colds—have previously been common understandings around sniffling. However, as Covid-19 spread globally, the contextual meaning of a runny nose changed (radically). A runny nose was still a runny nose; however, the pandemic discourse that evolved now added something more to it—an additional and/or competing meaning. Still, old “truths” remained. Drawing on our interviews, the sniffling could be understood, explained or repeated as an ordinary cold, “morning runny nose”, Covid-19 or pollen allergy, due to various considerations and agendas. To some extent, the existence of different possible truths made resistance without a clear intent possible.

The idea of intention, as framed in previous research, indicates that the minds of the resisters remain unpersuaded by hegemonic arguments and that subjects are free to take any action that they decide upon (Butz, 2015; Mitchell, 1990, pp. 562, 564). As Susan Gal (1995, pp. 420–421) points out, Scott uses a simple representational theory of language, in which the world and its realities are already in place, unmediated and unaffected by how it is narrated or constructed in language (Gledhill, 2000). In this storyline, resisting subjectivities are largely unaffected by dominating discourses. However, when reviewing our interviews, it appeared as both the resistance against power and the reasons for resisting were informed by—but not decided by—discourses that form subjectivities, truth regimes and realities (Baaz et al., 2017; Lilja & Öjendal, 2009). In addition, the quotations above indicate that there was some room to maneuver regarding how the subjects reflected and embarked upon different and often muddled discourses. Our respondents’ actions could be understood as an often involuntary and covert resistance that countered mainstream views on runny noses. This is a kind of micro-resistance, which is individual, localized and temporary, and should not be underestimated given that a multitude of acts of resistance can “set off an avalanche” (Scott, 1990, p. 192). Or as displayed by Mumby et al. (2017), minor resistance acts are sometimes the catalyst for larger changes.

The elaborated resistance can be better understood if we, as underscored by Ortner, abandon the simple opposition between “domination” and “resistance”. As Ortner argues, resisting subjects are sometimes ambivalent about what they are doing and why they are doing it (Ortner, 1995, p. 187). Notions of compliance and factual resistance co-exist in the acts and narratives of the sms-employees. Thus, as pinpointed by the Comaroffs and Comaroff, (1992), much of what goes on within power relationships and, as we suggest, the corresponding resistance practices, has a “murky” quality (Comaroff & Comaroff, 1992, p. 259; Gledhill, 2000).

While breaking state recommendations with, what could be understood as involuntary resistance, the sms-employees we interviewed did not participate in any resistance movements, and they sometimes collaborated with the local management (Ortner, 1995, p. 179; Gledhill, 2000). In fact, the sms-employees sometimes found that their rule-breaking was forced upon them by the local management, who encouraged the sick to be present at work. One sms-employee stated:

“Yes, now I have to go and do a corona test, because I'm worried about my symptoms”. Then it was almost the head of the unit who just said: “No, but you don't have to, do you?” Like this [laughs]. Because they want staff then. Or they just, “do you really feel sick?” I just “yeah, no, obviously I don't. But I have a stuffy nose and a runny nose. How the hell am I supposed to…? Should I hope it's not corona or not?” You know this way.

The involuntary resistance of our respondents was then facilitated, or even encouraged, by the local management. The resistance of the temporary employees shows that the idea of intent, in Scott’s sense, is not applicable. Rather, our respondents’ resistance was justified by existing discourses and was spurred by material conditions. It might be resistance that one does not think of as resistance and/or might think of differently later. Our respondents seemed aware that their presenteeism might be questioned. An illuminating example of this is that those who went to work sniffling, while rationalizing it as an allergy, still seemed to be aware of breaking the rules, therefore hiding it: “it was also a bit taboo. You know, you do not want to go to work having a running nose”.^32^ While knowing that their acts were deviating from the pandemic recommendations, their aim was not, however, to unleash any resistance against this advice, which they rather seemed to respect. Thus, the resistance can be regarded as involuntary and emerged from, what we label as, “complex intentions”.

As displayed above, the strong biopolitical and ethopolitical rule, which emerged during the Covid-19 pandemic, took place in a society that was marked by neoliberal norms and attached modes of subjectivation. The disciplining of colleagues interacted with the state recommendations in combination with a neoliberal management of working life. As heterogeneous elements came together—neoliberal management, state politics, local management, disciplinary punishments and hierarchical norms (based on class, sex and ethnicity)—neoliberal norms limited the possibilities of the ethopolitical rule of the state (at least in regard to our respondents). This, we would like to propose, tells us something about how multiple forms of governing can, at times, become frictional and create unexpected forms of resistance. In this case, the resistance can be considered involuntary; while our respondents considered the recommendation of the state reasonable, their actions still undermined them. They understood that they went against prevailing recommendations of the state; thus, there was some intention. However, in their understanding, they did not really challenge the advice as they did not consider themselves to have covid (but could have “morning runny nose”, an allergy or likewise). For our respondents, the reason for going to work seemed to be economic vulnerability, and their decision was supported by local managers, who at times encouraged presenteeism. Overall, intention and (un)intention entangled to form a complex mess.

## Concluding Discussion

“Intent” is a contested concept that has divided the field of Resistance Studies for (too) long. In this paper, we have elaborated on the concept in relation to “resistance” through a micro-study of the Covid-19 situation in Sweden during 2020 and 2021. Our respondents’ accounts show that for them, multi-layered forms of power and conflicting governing strategies at different levels of society turned into a seedbed for involuntary resistance. Community and state governing, and neoliberal norms were the context of the strong disciplinary governing that occurred in the wake of the Covid-19 pandemic in Sweden. These forms of governing interacted with other relations of power, such as hierarchies that were based on, for example, age, class and sex, which together created a specific form of resistance. Thus, we would like to argue that the framing of involuntary resistance illuminates the co-existence of different forms of governing, change processes and frictional neoliberalism.

The “involuntary” resistance addressed in this paper emerged from “muddled” intentions; that is, from a complex mix of both intention and non-intention. The sms-employees narrated some kind of intent but not the intent to resist power. The resistance could be seen as an effect of power(s), but was still accomplished without the aim to challenge power. In addition, the act of dissent involved some “rationale shopping”; for example, our interviews reveal how competing stories around “runny noses” created some room to maneuver. By choosing one storyline over others, the resisters took the decision to break the recommendations, which in practice meant that they refused to be called to order by the “ethopolitics” of the governing of the Swedish state.

To understand how resistance sometimes “accidentally” challenges power and reverses hierarchies, every case of resistance performance should be seen as a unique case that deserves to be further interrogated, analyzed and mapped. In this, it is important to remember that “resistance is not an intrinsic quality of an act but a category of judgment about acts” (Barker, 2004, p. 178). And so is intent. As stated by Scott in a recent text, it is not only the resisters’ intent that matters, but so does the intent of the audiences: “The poacher may be only interested in rabbit stew but when all his neighbors see it as a just use of the common lands, then it becomes, socially, an act of everyday resistance” (Scott, 2019, p. xi). From this perspective, intent, when constructed and located by others, can “make” non-intentional acts into acts of resistance. This is the kind of resistance that is illuminated in this paper.

## Footnotes

1 We have taken measures to protect our respondents. First and foremost, the names of the respondents will not be revealed, but the respondents have been given fictitious names.

2 https://www.folkhalsomyndigheten.se/smittskydd-beredskap/utbrott/aktuella-utbrott/covid-19/skydda-dig-och-andra/om-du-planerar-att-resa/.

3 https://www.folkhalsomyndigheten.se/the-public-health-agency-of-sweden/communicable-disease-control/covid-19/.

4 https://www.folkhalsomyndigheten.se/publicerat-material/publikationsarkiv/h/how-to-wash-your-hands/.

5 https://www.thelocal.se/20201122/in-english-prime-minister-stefan-lfvens-televised-speech-to-the-nation/.

6 https://www.thelocal.se/20200322/in-english-prime-minister-stefan-lfvens-address-to-the-nation/.

7 https://sverigesradio.se/artikel/7440302/.

8 https://www.aftonbladet.se/ledare/a/50qmd6/anders-tegnells-budskap-ar-att-vi-kan-tanka-sjalva/.

9 https://www.riksdagen.se/sv/dokument-lagar/dokument/protokoll/protokoll-20192095-fredagen-den-27-mars_H70995 /html/. The quotation is translated into English by the authors.

10 Interview in Swedish with a union official and assistant nurse (Amelia), Sweden 2021. The quotation is translated into English by the authors.

11 https://www.oecd.org/sweden/OECD-Income-Inequality-Sweden.pdf/.

12 https://www.scb.se/en/finding-statistics/statistics-by-subject-area/household-finances/income-and-income-distribution/income-and-tax-statistics/pong/statistical-news/income-mobility-20002020/.

13 Interview with an assistant nurse, who also worked as union official, (Amelia), Sweden 2021. The quotation is translated into English by the authors.

14 Interview in Swedish with a union official/assistant nurse (Amelia), Sweden 2021. The quotation is translated into English by the authors.

15 Interview in Swedish with a union official/assistant nurse (Amelia), Sweden 2021. The quotation is translated into English by the authors.

16 https://www.gp.se/debatt/de-otrygga-anställningarna-inom-omsorgen-måste-bort-1.32423196/. The quotation is translated into English by the authors.

17 https://www.gp.se/debatt/de-otrygga-anställningarna-inom-omsorgen-måste-bort-1.32423196/. The quotation is translated into English by the authors.

18 Interview in Swedish with a union official/assistant nurse (Cecilia), Sweden 2021. The quotation is translated into English by the authors.

19 Interview in Swedish with a person employed by the hour (Tove), Sweden 2021. The quotation is translated into English by the authors.

20 For a more extensive account of how pre-existing, structural conditions contributed to limiting the effectiveness of some governing interventions and how disadvantaged groups experienced disproportionate impacts both disease and of the measures introduced to manage it, see e.g., Blundell et al., 2020; Boin et al., 2021; Jasanoff et al., 2021.

21 https://lakartidningen.se/opinion/debatt/2020/04/covid-19-hos-personal-pa-boende/.

22 https://www.lo.se/home/lo/res.nsf/vRes/lo_fakta_1366027478784_vi_som_inte_jobbade_hemma_ pdf/$File/Vi_som_inte_jobbade_hemma.pdf/.

23 https://www.gp.se/debatt/de-otrygga-anställningarna-inom-omsorgen-måste-bort-1.32423196/.

24 Interview in Swedish with a union official/assistant nurse (Cecilia), Sweden 2021. The quotation is translated into English by the authors.

25 Interview in Swedish with a union official/assistant nurse (Amelia), Sweden 2021. The quotation is translated into English by the authors.

26 Interview in Swedish with a union official/assistant nurse (Amelia), Sweden 2021. The quotation is translated into English by the authors.

27 Interview with a person employed by the hour (Sam), Sweden 2021. The quotation is translated into English by the authors.

28 https://www.dagensmedicin.se/alla-nyheter/kommunal-halsa/gick-till-jobbet-med-covidsmitta-flera-atal/.

29 https://www.lo.se/home/lo/res.nsf/vRes/lo_fakta_1366027478784_vi_som_inte_jobbade_hemma_ pdf/$File/Vi_som_inte_jobbade_hemma.pdf/.

30 Interview with a union official/assistant nurse (Amelia), Sweden 2021. The quotation is translated into English by the authors.

31 Interview with a union official/assistant nurse (Amelia), Sweden 2021. The quotation is translated into English by the authors.

32 Interview with a person employed by the hour (Sofia), Sweden 2021. The quotation is translated into English by the authors.
